# Time for transplant care professionals to face recipients' fear of graft rejection—an opinion paper

**DOI:** 10.3389/frtra.2023.1277053

**Published:** 2023-09-29

**Authors:** Anna Forsberg, Nichon Jansen, David Paredes, Hannah Maple

**Affiliations:** ^1^ Institute of Health Sciences at Lund University, Lund, Sweden; ^2^ Department of Cardiothoracic Surgery, Skane University Hospital, Lund, Sweden; ^3^ Chair of European Transplant Allied Healthcare Professionals (ETAHP), a Section of the European Society for Organ Transplantation, Padua, Italy; ^4^ Policy Department, Dutch Transplant Foundation, Leiden, Netherlands; ^5^ Past-Chair of European Donation and Transplantation Coordination Organization (EDTCO), a Section of the European Society for Organ Transplantation, Padua, Italy; ^6^ Donation and Transplant Coordination Unit, Hospital Clinic, Associate Professor Surgical Department, University of Barcelona, Barcelona, Spain; ^7^ Department of Transplantation, Guy’s and St. Thomas’ NHS Foundation Trust, London, United Kingdom; ^8^ Chair of Ethical, Legal, and Psychosocial Aspects of Transplantation (ELPAT), a Section of the European Society for Organ Transplantation, Padua, Italy

**Keywords:** graft rejection, organ transplantation, psychological well-being, uncertainty, lived experience, transplant recipient

## Introduction

Transplantation is an established treatment that is constantly evolving and developing. Knowledge about patients' reactions to transplantation is a necessary foundation for high quality, person-centered care. When Organ Transplant Recipients (OTRs) are asked about what they fear the most, the most common answer is graft rejection (1–7). However, little attention has been paid to this concern since OTRs' perceptions and experiences, as well as consequences e.g., health-related quality of life, in relation to the fear of graft rejection have until now been poorly understood. There is only one validated domain-specific instrument to measure this perceived threat among OTRs receiving various types of solid organs (8). The absence of systematic and structured measurements limits the ability to make comparisons between groups of OTRs and to evaluate the effects of various interventions; something which in important for health care professionals (HCPs) when wishing to address this issue. Lack of awareness among HCPs might also be a barrier, leaving the OTR alone with their fears.

A survey launched by the European Society for Organ Transplantation (ESOT) in 2020 asked the European Transplant Patient Organizations (ETPO) about their five main concerns relating to transplantation. This demonstrated that the top concern above all others was fear of graft rejection and uncertainty regarding how long the graft will last (9). A collaborative project between ESOT and ETPO was conducted as part of the 2019 ESOT congress in Copenhagen. This novel alliance reemphasized the need for patients' perspectives regarding Perceived Threat of the Risk of Graft Rejection (PTRGR) after Solid Organ Transplantation (SOTx) to be taken seriously and addressed in everyday clinical practice and as part of long-term follow-up. Thus, the aim of this opinion paper is to raise awareness among HCPs regarding this topic from the perspective of the organ transplant recipients and provide an outline of interventions to inform and guide HCPs on this matter.

## Background

### Definitions and characteristics of risk, harm, and threat

To intervene as HCPs, it is vital that we understand the specific characteristics of PTRGR which is defined as “*the anticipation of graft rejection based on symptoms, signs, or the cognitive appraisal of graft rejection. This anticipation can elicit the stress response of organ transplant recipients, expressed as intrusive anxiety and fear of negative health implications for the future*” (10).

Risk has been defined in a concept analysis as being exposed to the likelihood of a negative event and to be an “at-risk person” means to be unintentionally at risk (11). OTRs are constantly exposed to the risk of graft rejection perceived as a negative event and consequently, are treated with immunosuppressive medication as long as the graft is in place. They are all informed that adherence to the immunosuppressive medication is vital for preventing graft rejection. Threat is described by Lazarus & Folkman as “*harms or losses that have not yet taken place but are anticipated*”, i.e., PTRGR. However, these are also present and persist once a harm or loss has been actualized by means of an actual graft rejection due to “*negative implications for the future*” (12). Threat can also be defined as being “*a threatening encounter that makes one feel uneasy (anxious), which is connected with a strong effort to protect oneself from anticipated danger*” (13). An example of this strong effort is being adherent to the immunosuppressive medication (14). Perceived threat is defined by Carpenter as “*the anticipation of harm that is based on the cognitive appraisal of an event or cue that is capable of eliciting the individuaĺs stress response*” (15). In relation to PTRGR it means an elevated and intrusive anxiety based on a constant anticipation of harm due to graft rejection. Harm relevant to the phenomenon of graft rejection may manifest as anticipated graft loss, interference with needs or goals and perceived loss of control in everyday life. It is important to acknowledge that concerns about graft rejection may be present even if graft survival is gradually improving. The potential for graft rejection might perceived as an ever-present threat (3), as expressed by a Liver Transplant Recipient at his one-year follow-up (LTR):

“*Sometimes you think about how long it all will last. For how long will a transplanted liver work? Those moments come and I [can] actually be sad… To get some extra time. I can have quality of life, despite living overtime. The difference between me and others is that we all have the return ticket booked, but mine is already printed out. I often think about that*” (16).

### Perceptions among adults

The perceptions of harm among adult SOTRs, explored by a phenomenographic research method (14) have been incorporated into five domains: (1) Abstract threat to life; (2) Concrete threat to health; (3) Trust in the body; (4) Striving to control the threat; (5) One's identity. Across these domains SOTRs varied in their views of graft rejection, from something manageable to a condition leading to serious illness and death. In “*abstract threat to life*” the risk of graft rejection was perceived as a constant threat. “C*oncrete threat to health*” revealed that blood tests and procedures (such as a biopsy) led to a high level of emotional stress due to fear that graft rejection may be confirmed. The increase in immunosuppressive medications also acted as a reminder. In the “*trust in the body*” domain the SOTRs utilized this approach as a means of gaining control over the threat. “*Striving to control the threat*” involved developing various strategies for mastering the process, based on the perception that graft rejection was something controllable. The domain “*one's identity*” involved their own perceptions of their ability to master the situation and being empowered to act upon experience-based knowledge (14). It was about viewing oneself as a capable person after going through a graft rejection and learning from that experience.

### Perceptions among children and adolescents

The perceptions of harm among adolescents, also explored using phenomenography comprise seven domains: (1) Tests and examinations; (2) The transplant; (3) Medication; (4) Graft rejection as a condition; (5) Graft rejection and its consequences; (6) Friends; (7) Oneself as an organ transplant recipient. “*Tests and examinations*” revealed a need to check the graft's function and the importance of blood tests as an indicator of health status. In the domain “*the transplant*” there was a basic understanding that the transplant was something inevitable and “*medication*” revealed how important this was within daily life. “*Graft rejection as a condition*” involved varied perceptions, indicating that rejection is considered as something insignificant, not worthy of attention and something that none of the adolescents had heard of. When asked about the concept of “*graft rejection and its consequences*”, views were in line with a more biomedical explanation, such as a deterioration leading to graft loss, the need for dialysis, hospitalization and, in the worst-case, re-transplantation. The domain “*friends*” indicated that friends and classmates, were familiar with their condition and situation, whilst “*oneself as an organ transplant recipient”* focused on normality and the normality of life (17). In a study involving children with liver transplants (aged between seven and fifteen), the major phenomenon was striving for normality, not thinking about graft rejection, and living in the same way as healthy children. The results also demonstrated that these children focused on events that supported the idea of being normal (18) and satisfaction with life meant being able to live a normal life, feeling better than before the transplant and to do almost everything they wanted (19). The key differences between adults and adolescents seem to be the awareness of graft rejection as something very serious among adults and something unknown and insignificant among adolescents. Also, the social context was emphasized by the adolescents while not significantly highlighted by the adults.

### Consequences

Although recipients are informed before their transplant to expect at least one episode of rejection, they are nevertheless surprised and frightened when it occurs (5). Anticipatory anxiety may precede the first rejection episode (20). However, once the routine for anti-rejection therapy is mastered, OTRs are generally more at ease. Graft rejection is often accompanied by withdrawal, depression and reactivation of feelings associated with previous health impairment (20), and depression might be associated with early rejection or infection (21). Knowledge of laboratory evidence of transplant graft malfunction may result in anger vented towards the surgical team and nursing staff for not doing enough to prevent a rejection event which may result in the patient's death (22). To summarize, OTRs expect damage to happen if graft rejection occurs with subsequent reduced function of their transplanted organ. The risk of graft rejection is perceived as an everlasting present threat affecting everyday life to some. The threat is related to fears of facing death, being as ill like before the transplant and losing their health and facing re-transplantation. The level of fear increases with procedures and whilst awaiting blood results. Efforts to cope with the risk of graft rejection, (i.e., the anticipated harm) vary and involve various efforts to protect oneself from this (17).

### Fear of graft rejection among different organ recipients along the course of transplantation

For more than two decades a Swedish research group has focused on recipients’ experiences of the specific event of graft rejection within the context of SOTx (5, 8, 10, 14, 16, 17, 23). OTRs expect damage to happen if graft rejection occurs, i.e., reduced function of their transplanted organ. One year after transplantation, the emotions of liver recipients towards the threat of graft rejection fluctuated between perceiving it as something of no specific concern or significance, to something associated with a fear of death. These feelings included recipients being constantly aware of their bodies, having a continual sense of fear, experiencing an invisible threat, and being failed or simply “let down” by their bodies in terms of the own immune system not accepting the new organ (5). Most OTRs make strong efforts to protect themselves from graft rejection e.g., taking their medication as strict as possible (14), and around 33% fear that it will occur (8). When investigating graft related threats in kidney, liver, heart or lung recipients, patients' scores were widely spread with 33%, 40% and 27% perceiving low, uncertain or high levels of graft related threat, respectively (8). However, there were few lung recipients in the study by Nilsson et al., and those who participated were included with heart recipients. The majority of the OTRs (74%) reported low levels of intrusive anxiety and a high level of lack of control (48%) which means that they don't think they can affect graft rejection (item 10–12 in the instrument displayed in Table 1) (8).

**Table 1:** The instrument perceived threat of the risk for graft rejection, (PTRGR).

		Strongly disagree	Disagree	Not sure	Agree	Strongly agree
1	Graft rejection means that my basic disease will return.	1	2	3	4	5
2	Graft rejection means that I will be as ill as before transplantation.	1	2	3	4	5
3	Graft rejection means losing my graft.	1	2	3	4	5
4	I think of graft rejection every day	1	2	3	4	5
5	I think of graft rejection whenever I take my medication.	1	2	3	4	5
6	I fear graft rejection when I await my laboratory results.	1	2	3	4	5
7	Nothing can distract me from worrying.	1	2	3	4	5
8	I experience great fear of how it will end.	1	2	3	4	5
9	Graft rejection is almost always on my mind.	1	2	3	4	5
10	I can’t affect graft rejection personally.	1	2	3	4	5
11	I can’t affect how it will turn out to be.	1	2	3	4	5
12	I doubt that I can do anything about this.	1	2	3	4	5

Items 1–3 measures the domain Graft Related Threat (GRT). Items 4–9 measures the domain Intrusive Anxiety (IA) and items 10–12 the domain Lack of Control (LOC).

There are extensive variations in perceptions of the risk of graft rejection among both adult and adolescent OTRs (17). Among adults the perceptions varied from involving the threat of dying to nothing particular to worry about, while among adolescents the main perception about graft rejection was that it was something completely unknown or just vaguely familiar (Table 2). What is important to appreciate is that it is the SOTRs perception of the cue or event that is meaningful, not the kind or quality of the perceived anticipated harm.

**Table 2:** Domains illustrating perceptions of experiences of fear of graft rejection among adults (*n* = 16) and adolescents (*n* = 8).

Adults’ perceptions	Adolescents’ perceptions
The abstract threat to life The concrete threat to health Trust in the body Striving to control the threat One's identity	Tests and examinations The transplantation Medication Graft rejection as a condition Graft rejection and its consequences Friends Oneself as an organ transplant recipient

The adults were transplanted with kidney, liver, heart or lungs, aged 21–63 years and with a follow-up between three months and 10 years. The adolescents had received either a kidney or a liver, were aged 13–18 years with a follow up between five months and 14 years.

After transplantation, lung recipients strive for normalcy (7) and to find a new normality after transplantation. Striving for normalcy was the core process involved in the symptom experience and interpretation of graft rejection. Normality was also a common theme in studies that focused on adolescent organ transplant recipients (18, 19). In a recent study of lung recipients (*n* = 117) it was revealed that (PTRGR) expressed as a graft related threat, intrusive anxiety and lack of control was low 1–5 years after transplantation with no gender differences (24). Lung recipients aged over 50 years reported a higher level of intrusive anxiety than younger recipients. Although the overall prevalence of problematic intrusive anxiety was low, it explained close to 25% of the variance in general psychological well-being. Among 79 heart recipients, the majority (72%) reported low graft related threat whilst the remainder perceived graft rejection as a serious threat. Intrusive anxiety was low. Thirty-seven percent believed that the threat of graft rejection was beyond their control, and they were unable to protect themselves from it, thereby suggesting a fatalistic approach (25). Heart recipients with high level of fatigue and low psychological well-being reported stronger intrusive anxiety and less control. Further, those with a history of at least one self-reported rejection episode experienced less graft related threat. When exploring how OTRs learn about graft rejection (14) almost half of the informants had experienced a rejection episode. Those who had experienced symptoms at the time of rejection paid attention to signals from their body, searched for information among other OTRs and learned from the latter's experiences. The opposite approach was avoidance where the patients that had experienced graft rejection did not search for information and avoided discussing the matter with other patients. Regardless of approach both groups felt safe and comfortable with their health situation since they had experienced that a graft rejection is manageable. We hypothesize that this is due to a positive experience of successfully treating graft rejection, which increases the trust in the organ and/or reduces the perceived threat.

A systematic review (26) on research priority setting in organ transplantation revealed that stakeholders (i.e., patients, caregivers, living kidney donors and health professionals) identify graft-related complications as an important research area. Topics include graft function, and acute and chronic rejection. In conclusion, prevalence of fears associated with graft rejection among different SOTRs is well known, as well as its relationship with psychological well-being. Now is the moment to address these fears as HCPs and act towards helping our SOTRs overcome them. For this purpose, a theoretical framework might be helpful.

## A theoretical framework for interventions and measurements

### The theory

A good starting point for a theory driven approach towards the fear of graft rejection is the theory of PTRGR (10). The meta-concepts of the theory are: Person, Health, Environment and Caring actions. Person refers to an OTR being subjected to lifelong immunosuppressive medication because of a constant risk of graft rejection, so to prevent the harms associated with graft rejection should these medications not be taken. Health means the need to experience comfort and cognitive understanding to master the PTRGR, resulting in life satisfaction. The environment is any context where the OTR is trying to master his or her everyday life. Caring actions involve either subjective or objective assessments by the HCP as a means of performing context-specific deliberate actions to approach and assess threat-induced emotions, and to relieve intrusive anxiety in the OTR.

### Benefits of a framework

The purpose of such a theory is to assist HCPs caring for OTRs who are suffering from threat-induced emotions due to the constant risk of graft rejection, such that their everyday lives are limited and negatively affected. It can also help detect risky, protective behaviors adopted by the OTR to prevent graft rejection or manage the sense of fear, which includes behaviors such as isolation, avoidance, and non-adherence. It is the only transplant specific theory available which focuses on the fear of graft rejection and provides a detailed interventional framework for HCPs to assist OTRs experiencing profound fears that affect their everyday lives.

Further actions include the development of person-centered care plans, implementation of threat reducing interventions that promote the SOTRs' mastering of graft-related threat and support to adopt useful and reasonable strategies to protect oneself from the harm of graft rejection. SOTRs should be first evaluated by assessing the degree of perceived threat, with specific attention paid to intervening variables such as graft function, immunosuppressive regimen and its possible side effects, health literacy, graft-related coping strategies, barriers for medication adherence, and social support. Protective coping strategies adopted by the OTR to protect themselves are additional resources also worthy of consideration (10).

### Measurement

Transplant professionals can address the PTRGR by utilizing a validated instrument specifically designed to measure it, as a means of screening SOTRs during outpatient follow up. This instrument has been developed specifically to screen OTRs for graft related fear and is psychometrically evaluated (8). It includes 12 items on a 5-point Likert-type scale (Table 1). Exploring the topic with the PTRGR-instrument creates awareness and provides a platform for further assessment, and additionally normalizes the topic within transplant follow up consultations. “G*raft-related threat*” (GRT) is the perception that the primary disease will return, leaving one as ill as before the transplantation and facing re-transplantation. Thus, this factor shows the extent of the threat of anticipated harm and implications for the future. The total GRT-score ranges from 3 to 15, where a score greater than 9 indicates a strong belief that graft rejection is a serious threat.

The second factor, “*intrusive anxiety*” (IA) means being constantly aware of the risk of graft rejection and preoccupation with this thought. It also implies a heightened level of anxiety, which is elevated further when taking immunosuppressive medication or undergoing a biopsy. Thus, this factor shows the extent of the OTRs' stress response and level of anxiety. The total IA score ranges from 6 to 30, where a score greater than 18 indicates great intrusion. Finally, the third factor, “*lack of control*” (LOC) involves perceptions that the threat of the risk of graft rejection is beyond one's control, revealing the degree of belief that one can control and protect oneself from it. The total LOC score ranges from 3 to 15, where a score greater than 9 indicates the perception of low control. Inter-item correlation values for this instrument range from 0.72 to 0.89 and the Cronbach's Alpha ranges from 0.81 to 0.91 (17). The PTRGR-instrument can also serve as a patient reported outcome measure (PROM) (27).

### How the framework can guide interventions

**Figure 1:**
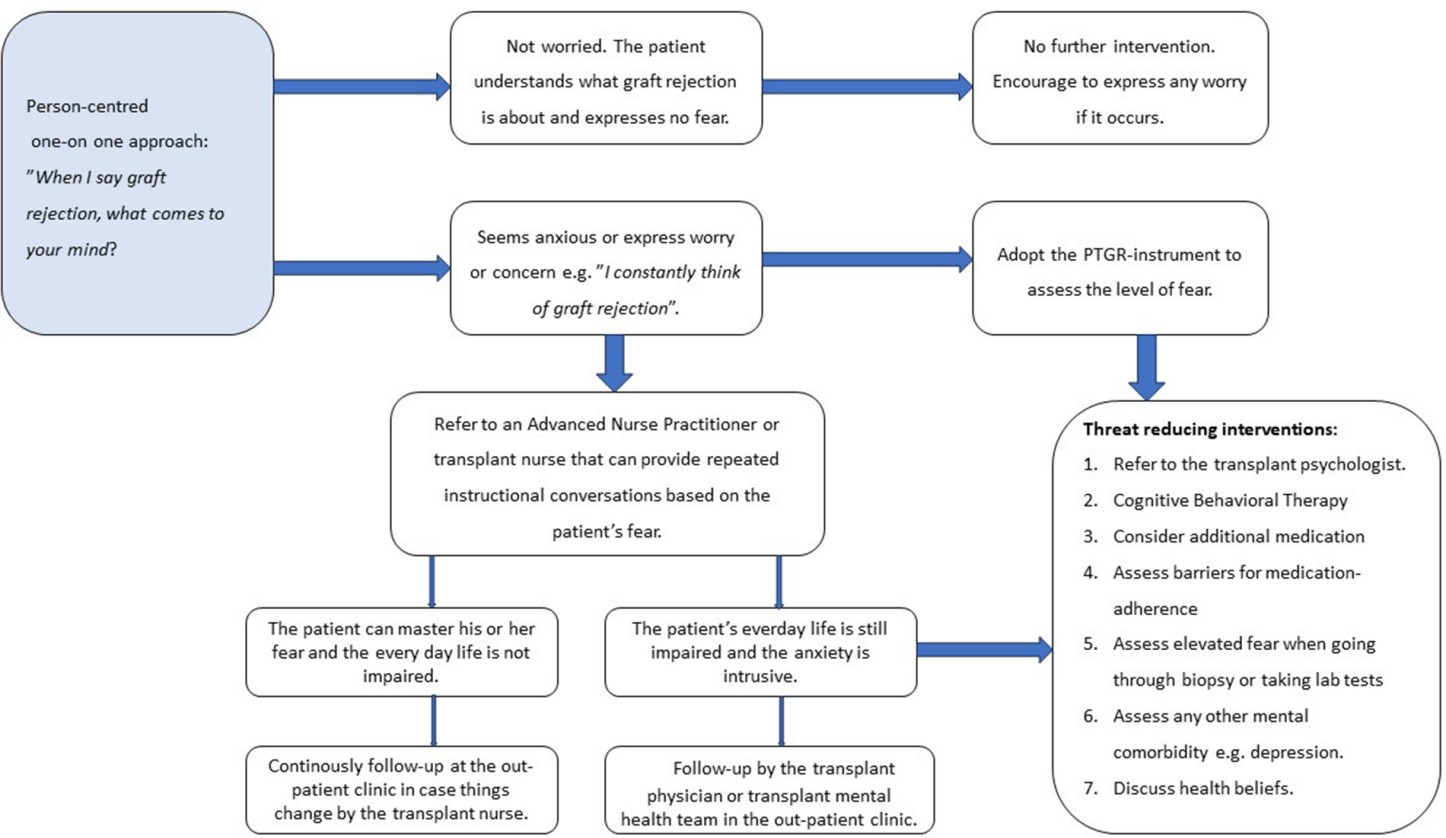
Clinical framework for assessment of fear of graft rejection and threat reducing interventions involving the multi-professional transplant team.

Given that the Theory of the Perceived Threat of the Risk of Graft Rejection (10) including the meta-concepts Person, Health, Environment and Caring actions, offers a solid interventional framework for threat reducing interventions, we propose the following approach to addressing this important topic as displayed by a flow chart (Figure 1).

• First approach the topic of PTRGR routinely in the outpatient clinic approximately three months after the transplant, once the demanding first phase of post-transplant recovery is over. Perform an initial assessment with a one-on-one approach, listening to the OTRs personal perceptions of experiences of graft rejection i.e., approaching the person with an organ. A simple introductory question might be the following: “When I say graft rejection, what comes to your mind”? This question constitutes the first step in the screening process and helps identify those with an intrusive fear. If the OTR says that he or she constantly thinks about graft rejection that might be a barrier for health.
• If the opening question exposes marked or profound fear within the OTR that is believed to be a barrier for life satisfaction (i.e., addressing the environment), we would recommend a systematic evaluation via the 12-item PTRGR-instrument described above. A score of 9 or above on the graft related threat scale (item 1–3) indicates a strong sense of graft related threat which might have a negative impact on the person's health as well as a score of 18 or above on intrusive anxiety (item 4–9) should be viewed as a concern. A score of 10 or above on the dimension lack of control (item 10–12) suggests a fatalistic approach.
• Where PTRGR is identified, the third step is to provide threat reducing caring interventions e.g., repeated instructional conversations to promote the mastering of a graft-related threat, specifically discussing the meaning of graft rejection at the follow-up visits. This could be scheduled with e.g., a psychologist, medical social worker or an Advanced Nurse Practitioner at the transplant follow-up clinic and take place whilst waiting for test results and medical consultation.
• Various methods of Cognitive Behavioural Therapy (CBT) [e.g., Acceptance and commitment therapy (ACT) or metacognitive therapy (MCT)] may also be used successfully by a psychologist in the transplant team (28).
• Finally, it is important to assess the level of fatigue and psychological wellbeing using appropriate instruments. This caring intervention is to evaluate the psychosocial consequences of PTRGR, and the uncertainty associated with it. It emphasises the need for person-centred education and mental support to be provided on a regular basis.

## Conclusion

This paper highlights the necessity of acknowledging the fear of graft rejection among SOTRs and provides theory driven guidance for HCPs in helping patients to deal with this fear. Organ transplantation is a significant achievement in modern medicine and healthcare. However, the reality for those being transplanted is that it affects every aspect of their life, mental and existential aspects in particular. A lot is known from the literature about the characteristics and prevalence of graft related fear. What is needed to help address this is a rigorous approach by transplant care professionals which is informed by discoveries in transplant related psychological or nursing research. This in turn will provide tools to help identify and help our patients deal with their fear of graft rejection.
